# Cucurbitacin I Attenuates Cardiomyocyte Hypertrophy via Inhibition of Connective Tissue Growth Factor (CCN2) and TGF- β/Smads Signalings

**DOI:** 10.1371/journal.pone.0136236

**Published:** 2015-08-21

**Authors:** Moon Hee Jeong, Shang-Jin Kim, Hara Kang, Kye Won Park, Woo Jin Park, Seung Yul Yang, Dong Kwon Yang

**Affiliations:** 1 College of Life Sciences, Gwangju Institute of Science and Technology, Gwangju, Republic of Korea; 2 Department of Veterinary Pharmacology and Toxicology, College of Veterinary Medicine, Chonbuk National University, Jeonju, Jeonbuk, Republic of Korea; 3 Division of Life Sciences, College of Life Sciences and Bioengineering, Incheon National University, Incheon, Republic of Korea; 4 Department of Food Science and Biotechnology, College of Biotechnology and Bioengineering, Sungkyunkwan University, Suwon, Republic of Korea; 5 Department of Horticulture, Sunchon National University, Sunchon, Jeonnam, Republic of Korea; University of Cincinnati, College of Medicine, UNITED STATES

## Abstract

Cucurbitacin I is a naturally occurring triterpenoid derived from Cucurbitaceae family plants that exhibits a number of potentially useful pharmacological and biological activities. However, the therapeutic impact of cucurbitacin I on the heart has not heretofore been reported. To evaluate the functional role of cucurbitacin I in an *in vitro* model of cardiac hypertrophy, phenylephrine (PE)-stimulated cardiomyocytes were treated with a sub-cytotoxic concentration of the compound, and the effects on cell size and mRNA expression levels of ANF and β-MHC were investigated. Consequently, PE-induced cell enlargement and upregulation of ANF and β-MHC were significantly suppressed by pretreatment of the cardiomyocytes with cucurbitacin I. Notably, cucurbitacin I also impaired connective tissue growth factor (CTGF) and MAPK signaling, pro-hypertrophic factors, as well as TGF-β/Smad signaling, the important contributing factors to fibrosis. The protective impact of cucurbitacin I was significantly blunted in CTGF-silenced or TGF-β1-silenced hypertrophic cardiomyocytes, indicating that the compound exerts its beneficial actions through CTGF. Taken together, these findings signify that cucurbitacin I protects the heart against cardiac hypertrophy via inhibition of CTGF/MAPK, and TGF- β/Smad-facilitated events. Accordingly, the present study provides new insights into the defensive capacity of cucurbitacin I against cardiac hypertrophy, and further suggesting cucurbitacin I’s utility as a novel therapeutic agent for the management of heart diseases.

## Introduction

Cardiac hypertrophy is an adaptive response of the heart to various pathological stimuli (e.g., hypertension, valvular disease, and myocardial infarction). The condition is characterized by the enlargement of cardiac myocytes, accumulation of sarcomeric proteins, and sarcomeric reorganization [1]. Although cardiac hypertrophy is thought to compensate for mechanical loading in its initial stages, sustained cardiac hypertrophy often proceeds to an advanced disease state. Therefore, cardiac hypertrophy is a major independent risk factor for cardiovascular morbidity and mortality [2].

Connective tissue growth factor (CTGF), also designed CCN2, is an extracellular matrix (ECM)-secreted protein of the CCN (*C*yr61, CTGF, and *N*ov) family of proteins [3]. CTGF displays multiple biological actions, participating in cell proliferation, cell adhesion, cell migration, and extracellular matrix production. In particular, CTGF is a key mediator and biochemical marker of tissue fibrosis [4]. Similarly, numerous studies have demonstrated that CTGF crucially contributes to the pathogenic process of cardiac fibrosis. CTGF is a pro-hypertrophic factor in cardiac myocytes. CTGF is up-regulated in cardiovascular diseases. Furthermore, CTGF activate numerous MAPKs, hypertrophic activators, including ERK1/2, JNK, and p38 kinases [5]. Additionally, various kinds of extracellular stimuli (e.g., TGF-β, endothelin-1, and VEGF) upregulate CTGF expression. Among them, TGF-β and CTGF have a cooperative interaction to elicit overt cardiac hypertrophy and fibrosis [6]. Subsequently, activated TGF-β propagates its downstream intracellular pro-hypertrophic signals through the activation of Smad proteins [7].

Recently, many naturally occurring, plant-derived compounds have been successfully employed in alternative strategies for the treatment of various disorders, encompassing cancers, inflammatory diseases, diabetes, and so on. In addition, a number of natural compounds are also potentially applicable for the management of cardiac hypertrophy, heart failure, cardiac infarction, and other heart diseases [8, 9]. Of these, resveratrol, a polyphenol found in grapes, soybeans, and red wine, is under intensive investigation for its beneficial actions in assorted animal models of cardiovascular diseases [10], as well as for its potent antioxidant and metabolic properties [11]. These protective effects are reportedly dependent upon activation of Sirt1 and AMP-activated protein kinase (AMPK) [12, 13]. Of note, Sirt1 is an essential regulator of vital metabolic processes, including lipolysis, fatty acid oxidation, mitochondrial biogenesis, and gluconeogenesis [11, 14, 15]. Therefore, we anticipate that novel natural compounds will provide excellent molecular foundations for the development of new cardiovascular therapeutics.

The cucurbitacins comprise a group of highly oxygenated triterpenoids originally isolated from Cucurbitaceae family plants, in addition to other plant types (i.e., cucumber, melon, watermelon, and pumpkin) [16, 17]. Up until now, more than 40 native cucurbitacins and their derivatives have been isolated [15], with cucurbitacin B, E, D, and I receiving special scrutiny due to their relative abundance in plants [16]. The cucurbitacins exhibit a wide range of biological and pharmacological actions, including anti-cancer, anti-inflammatory, hepatoprotective, antioxidant, and cytotoxic actions [18–21]. These actions are all mediated in part via distruption of the Janus kinase/signal transducer and activator of transcription 3 (JAK)/STAT3 signaling pathway, known for its important roles in tumorigenesis, inflammation, cell proliferation, and cell differentiation [22–24].

In the present study, we first demonstrated that cucurbitacin I significantly attenuated Phenylephrine (PE)-stimulated cardiomyocytes, which provide an *in vitro* model of cardiac hypertrophy. Cucurbitacin I also impaired CTGF, mitogen-activated protein kinase (MAPK), and transforming growth factor-β (TGF-β)/Smad signaling events in the hypertrophic cardiomyocytes. These observations uncover the prospective use of cucurbitacin I as a therapeutic agent for the treatment of cardiac diseases.

## Materials and Methods

### Animal models

All animal experiments in this study were approved by the Animal Care Committee of Gwangju Institute of Science and Technology (Approval number: GIST 2012-15) and were performed according to the guidelines from the GIST IACUC the NIH principles for the Care and Use of Laboratory Animals. All efforts were made to minimize suffering.

### Cell culture and hypertrophic stimulation with PE

Neonatal rat cardiomyocytes were obtained from 1-to 2-day-old Sprague-Dawley rats as described previously [25]. In brief, the ventricular tissue was removed and enzymatically dissociated, and the resulting cell suspension was enriched for cardiomyocytes by using step gradient of Percoll (Amersham Biosciences, Piscataway, NJ, USA) for density separation. Isolated cardiomyocytes were plated onto either collagen-coated culture dishes or coverslips and then cultured in cardiomyocyte culture medium consisting of DMEM supplemented with 10% fetal bovine serum, 1% antibiotics cocktail (15240-062), 2 mM L-glutamine and 100 μM 5-bromodeoxyuridine (GIBCO-BRL, Grand Island, USA) at 37°C under 5% CO_2_.

Cucurbitacin I was purchased from Sigma Chemical Co. (St.Louis, USA) and dissolved in dimethyl sulfoxide (DMSO; Sigma, St.Louis, USA). Neonatal rat cardiomyocytes were cultured in serum-free medium for at least 24 h, after which time they were treated with cucurbitacin I for the cell viability assay, as described below. Alternatively, the cardiomyocytes were pretreated with cucurbitacin I (1 μM) for 24 h, followed by exposure to PE (100 μM) for the indicated time to induce hypertrophy.

### Cell viability assays

Cell viability was assessed by using the Cell Counting Kit-8 (CCK-8; Dojindo Laboratories, Kumamoto, Japan) assay. Briefly, neonatal rat cardiomyocytes were seeded into 96-well plates at a density of 2000 cells/well and treated with cucurbitacin I at 0.1, 0.5, 1, 5, and 10 μM in triplicate. After 24, 48, and 72 h, the CCK-8 reagent was added to the culture, and the cardiomyocytes were incubated at 37°C for an additional 4 h. Absorbance was measured at 450 nm by using a microplate reader.

### Immunostaining and cell size measurement

After experimental treatment with cucurbitacin I and/or PE, neonatal rat cardiomyocytes grown on collagen-coated cover slips were fixed with 4% paraformaldehyde for 10 min, permeabilized with 0.5% Triton X-100 in phosphate buffered saline for 10 min, and blocked with 5% bovine serum albumin for 1 h at room temperature. The cells were then incubated with a specific primary antibody against α-actinin antibody (1:200 dilution; A7811, Sigma) at 4°C overnight, followed by an Alexa 488-conjugated anti-mouse secondary antibody (1:200; 50968A, Invitrogen, Grand Island, NY, USA) for 1h at room temperature. Immunofluorescence staining was observed under a microscope equipped with a 40x objective lens and epifluorescence filters (Olympus Optical, Tokyo, Japan). Cell surface areas were measured using NIH imageJ software (available at http://rsb.info.nih.gov; National Institutes of Health (NIH), Bethesda, MD, USA).

### Quantitative real-time polymerase chain reaction (RT-PCR)

Total RNA was isolated from neonatal rat cardiomyocytes by using the TRI reagent (Sigma). To assess the mRNA expression levels of hypertrophic markers (ANF and β-MHC) and CTGF, reverse transcriptase reactions were performed by using ImProm II Reverse Transcriptase (Promega, Madison, WI, USA) with oligo-dT priming. Quantitative real-time PCR (qRT-PCR) was performed by using a TaKaRa Thermal Cycler Dice Real Time System Single TP 815 (Takara, Shiga, Japan) with SYBR Green (Takara, Shiga, Japan) as the fluorescent dyes. The primers were as follows: ANF forward: 5’-ACCTGCTAGACCACCTAGAGG-3’, ANF reverse: 5’- GCTGTTATCTTCCGTACCGG-3’; β-MHC forward: 5’-CAGACATAGAGACCTA CCTTC-3’, β-MHC reverse: 5’-CAGCATGTCTAGAAGCTCAGG-3’; CTGF forward: 5’-CAAGGACCGCACAGTGGTT-3’, CTGF reverse: 5’-GCAGTTGGCTCGCATCATAG-3’; and GAPDH forward: 5’-CTCTACCCACGGCAAGTTC-3’, GAPDH reverse: 5’-GCCAGTAGACTCCACGACATA-3’.

### Western blotting

Neonatal rat cardiomyocytes were treated with cucurbitacin I and/or PE, harvested, and lysed in RIPA buffer (1% NP-40, 50 mM Tris-HCl, pH 7.4, 150 mM NaCl, and 10 mM NaF) containing protease inhibitor cocktail (Roche Diagnostics, Manheim, Germany) and phosphatase inhibitor cocktail (Sigma). Protein homogenates were separated on SDS-PAGE gels and transferred to PVDF membranes (Bio-Rad Laboratories, Hercules, CA, USA). After blocking for 1 h with 5% non-fat dry milk, the membranes were incubated overnight at 4°C with antibodies against CTGF (1:1000; SC-14939, Santa Cruz Biotechnology), TGF-β (1:1000; SC-146, Santa Cruz Biotechnology, Dallas, TX, USA), extracellular regulated kinase1/2 (ERK1/2; 1:1000, CST-9102, Cell Signaling, Beverly, USA), phosphorylated ERK1/2 (p-ERK 1/2; 1:1000, CST-9101, Cell Signaling), c-Jun N-terminal kinase (JNK; 1:1000; CST-9252, Cell Signaling), p-JNK (1:1000; CST-9251, Cell Signaling), p38 (1:1000; CST-9212, Cell Signaling), p-p38 (1:1000; CST-9211, Cell Signaling), Smad2 (1:1000; CST-3103, Cell Signaling), p-Smad2 (1:1000; CST-3101, Cell Signaling), Smad3 (1:1000; CST-9523, Cell Signaling), p-Smad3 (1:1000; CST-9520, Cell Signaling), Smad7 (1:1000; 42-0400, Invitrogen), or GAPDH (1:2000; ab37168, Abcam, Cambridge, MA, USA). Next, the membranes were incubated with the appropriate HRP-conjugated secondary antibodies (1:10,000; rabbit, LF-SA5002; mouse, LF-SA5001; rat, LF-SA5003; AbFrontier, Seoul, South Korea) and developed by using a chemiluminescent substrate and an enhanced chemiluminescence kit (PerkinElmer, Waltham, MA, USA). Equal protein loading was confirmed by probing for GAPDH on the same membrane, and the intensity of each protein was quantified by using NIH ImageJ software.

### Transfection of small interfering RNA (siRNA) into cardiomyocytes

CTGF, TGF-β siRNAs and scrambled siRNA were purchased from Dharmacon, Inc. (Lafayette, CO, USA). Neonatal rat cardiomyocytes were cultured in serum-free medium for at least 24 h and each siRNA (50 nM) was then transfected into the cells by using lipofectamine 2000 (Invitrogen) according to the manufacturer’s protocol. After another 24 h, the cardiomyocytes were pretreated with cucurbitacin I, followed by exposure to PE for hypertrophic stimulation.

### Statistical analysis

All data are reported as the mean ± the SD. Statistical significance was analyzed by using the Student’s *t* test or a two-way analysis of variance (ANOVA) with a Bonferroni post-hoc analysis for multiple comparisons with the aid of Statview 5.0 software (SAS Institute, Cary, NC, USA). In all cases, *P* <0.05 was considered statistically significant.

## Results

### Cytotoxic actions of cucurbitacin I in cultured neonatal rat cardiomyocytes

**Fig 1:**
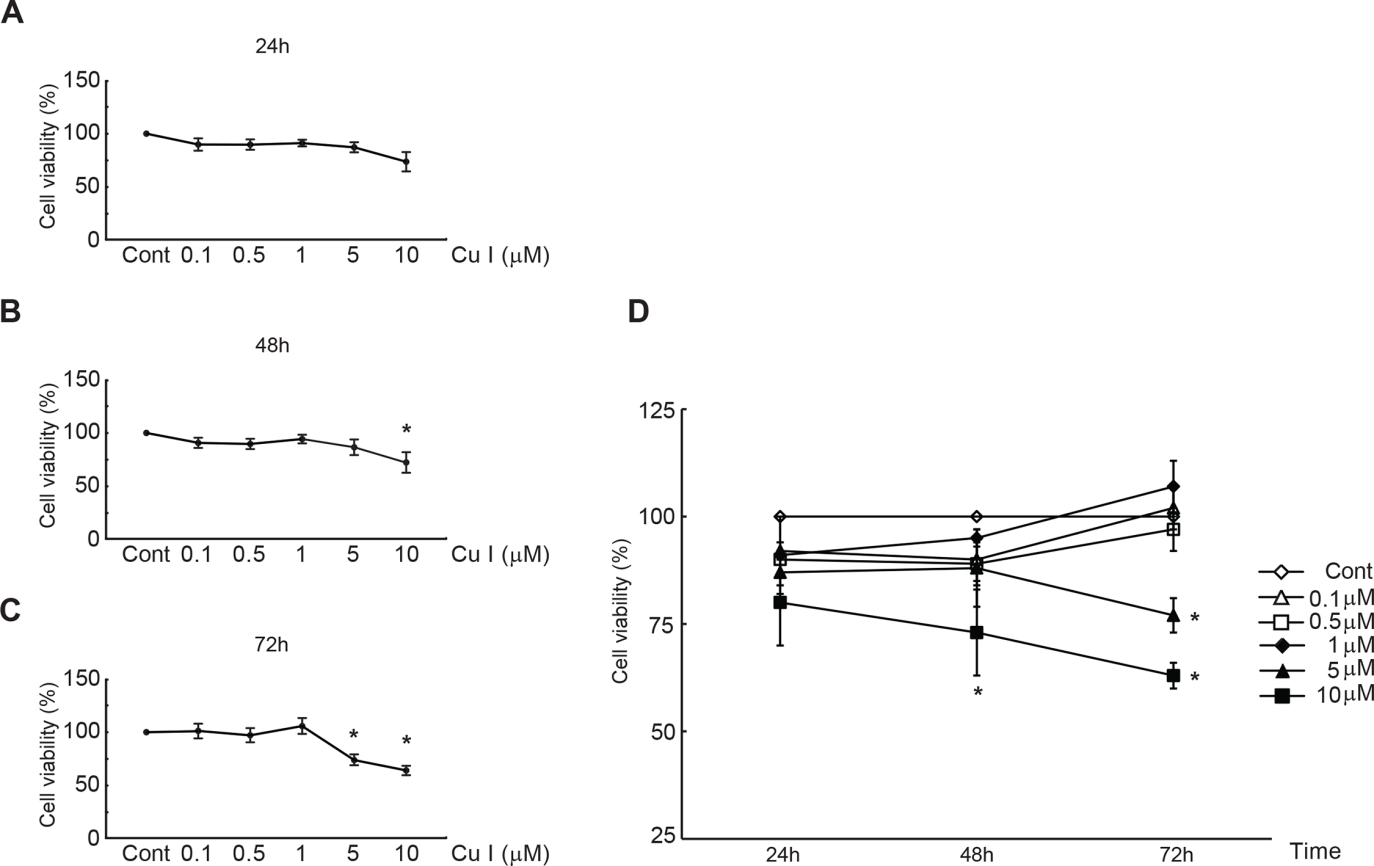
Cytotoxic effects of cucurbitacin I in cultured neonatal rat cardiomyocytes. Cell viability was measured in cardiomyocytes treated with the DMSO (control) or cucurbitacin I at 0.1, 0.5, 1, 5, and 10 μM for 24 h (A), 48 h (B), and 72 h (C). (D) Cell viability at each concentration on a time-dependent manner. Data are expressed as the means ± S.D. from three independent experiments. Significance was determined via a two-way ANOVA. *P < 0.05 vs. control group. Cont, control; Cu I, cucurbitacin I.

To test the cytotoxic effects of cucurbitacin I in cultured neonatal rat cardiomyocytes, the viability of the cells was investigated by employing the CCK-8 assay after treatment with 0.1, 0.5, 1, 5, and 10 μM cucurbitacin I for 24, 48, and 72 h. Exposure of the cardiomyocytes to cucurbitacin I at 0.1-1 μM for any length of time did not induce a significant decrease in cell viability (Fig 1A, 1B, 1C and 1D). However, cucurbitacin I at higher concentrations (5 and 10 μM) for 48 or 72 h triggered substantial cell mortality. For example, treatment with the compound at 5 μM for 48 and 72 h reduced cell viability to 86% and 74% of the value observed in control (DMSO-treated) cultures, respectively. Similar results were found for incubation of the cardiomyocytes with 10 μM cucurbitacin I treatment for 48 and 72 h, with cell viability reduced to 72% and 64% of the control value, respectively (Fig 1B, 1C and 1D). Therefore, cucurbitacin I was employed at 1 μM for further experimentation to assess its impact on hypertrophic responses in PE-stimulated cardiomyocytes.

### Cucurbitacin I attenuates hypertrophic responses in PE-stimulated cardiomyocytes

**Fig 2:**
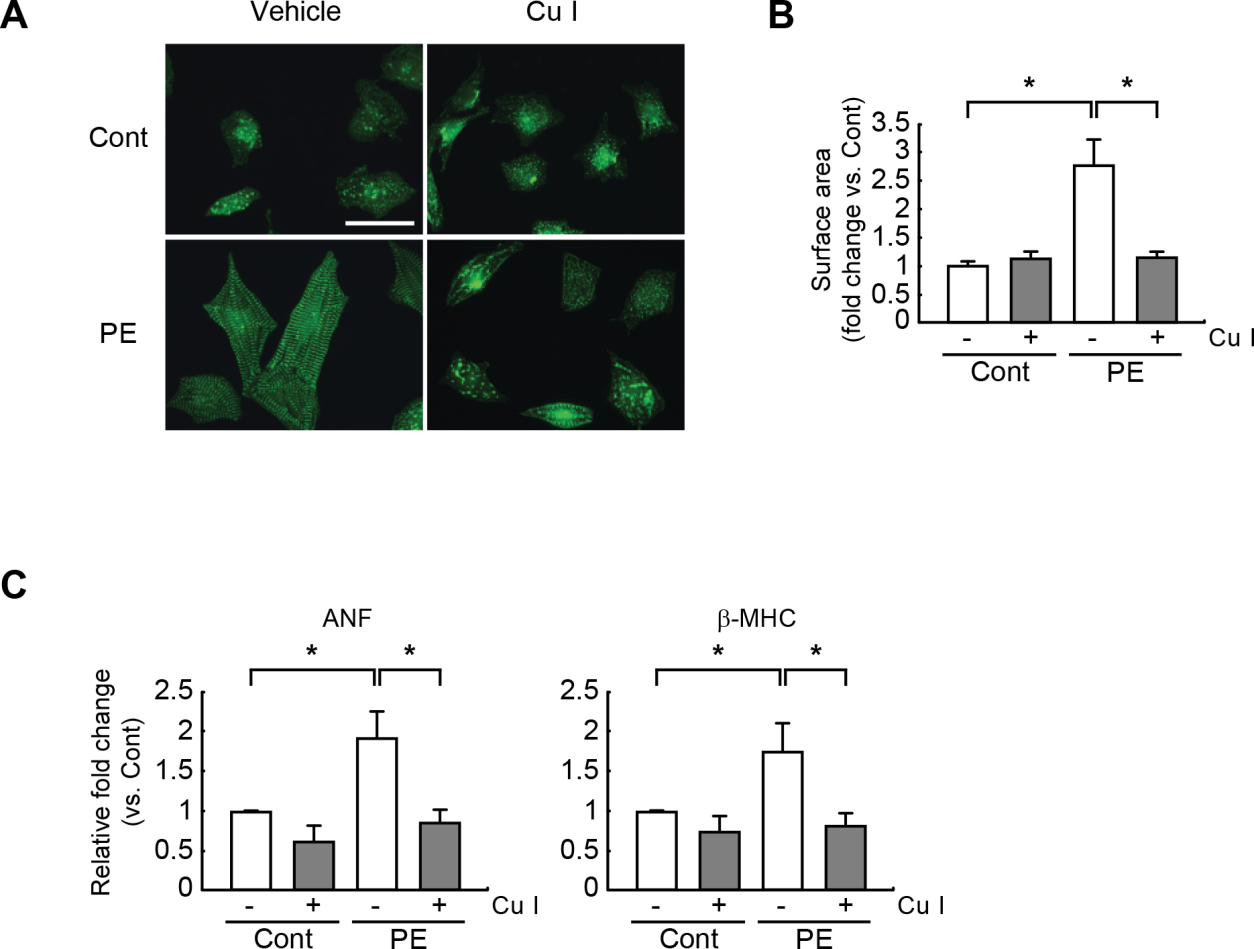
Cucurbitacin I attenuates hypertrophic responses in PE-stimulated cardiomyocytes. (A) Representative photograph of cardiomyocytes treated with PE (100 μM) after cucurbitacin I (1 μM) pretreatment. Sarcomeric organization of the cardiomyocytes was visualized by staining with an anti-**α**-actinin antibody. The sarcomeric response was completely blocked by pretreatment with cucurbitacin I. (B) Cell surface areas were measured by using NIH Image J software (n = 100 cells). Scale bars, 50 μm. (C) Quantitative RT-PCR analysis for ANF and β-MHC mRNA expression in cucurbitacin I-pretreated/PE-treated rat cardiomyocytes. The RT-PCR analysis was performed in triplicate with three independent samples. Data are expressed as fold changes ± S.D. vs. the control group. Significance was measured via a two-way ANOVA. * *P* < 0.05. Cont, control; Cu I, Cucurbitacin I.

To determine whether cucurbitacin I can alleviate cardiomyocyte hypertrophy, neonatal rat cardiomyocytes were cultured for 24 h with or without cucurbitacin I (1 μM). They were then further treated with PE (100 μM) for additional for 24 h. The hypertrophic response of cardiomyocytes is characterized by an increased cell size and a pronounced sarcomeric rearrangement, along with the induced expression of hypertrophic markers (e.g., ANF and β-MHC). Immunofluorescence staining with an α-actinin antibody revealed visibly larger cells with sarcomeric rearrangement in PE-treated vs. vehicle-treated control cardiomyocytes. Otherwise, cucurbitacin I-pretreated/PE-treated cardiomyocytes did not show the increase of cell size the sarcomeric rearrangement (Fig 2A). Moreover, quantification of the cell surface areas showed a significant (2.8-fold) increase in the size of the PE-treated vs. vehicle-treated control cardiomyocytes. However, this increase was significantly overturned by cucurbitacin I pretreatment of the PE-treated cells (Fig 2B).

Quantitative RT-PCR further indicated that the mRNA expression levels of ANF and β-MHC were elevated in PE-treated compared with control cells. Nevertheless, the increased expression levels were again significantly inhibited by pretreatment with cucurbitacin I (Fig 2C). Collectively, these results demonstrated that cucurbitacin I can prevent hypertrophic responses in PE-stimulated cardiomyocytes.

### Cucurbitacin I inhibits CTGF and MAPK signaling in PE-stimulated cardiomyocytes

**Fig 3:**
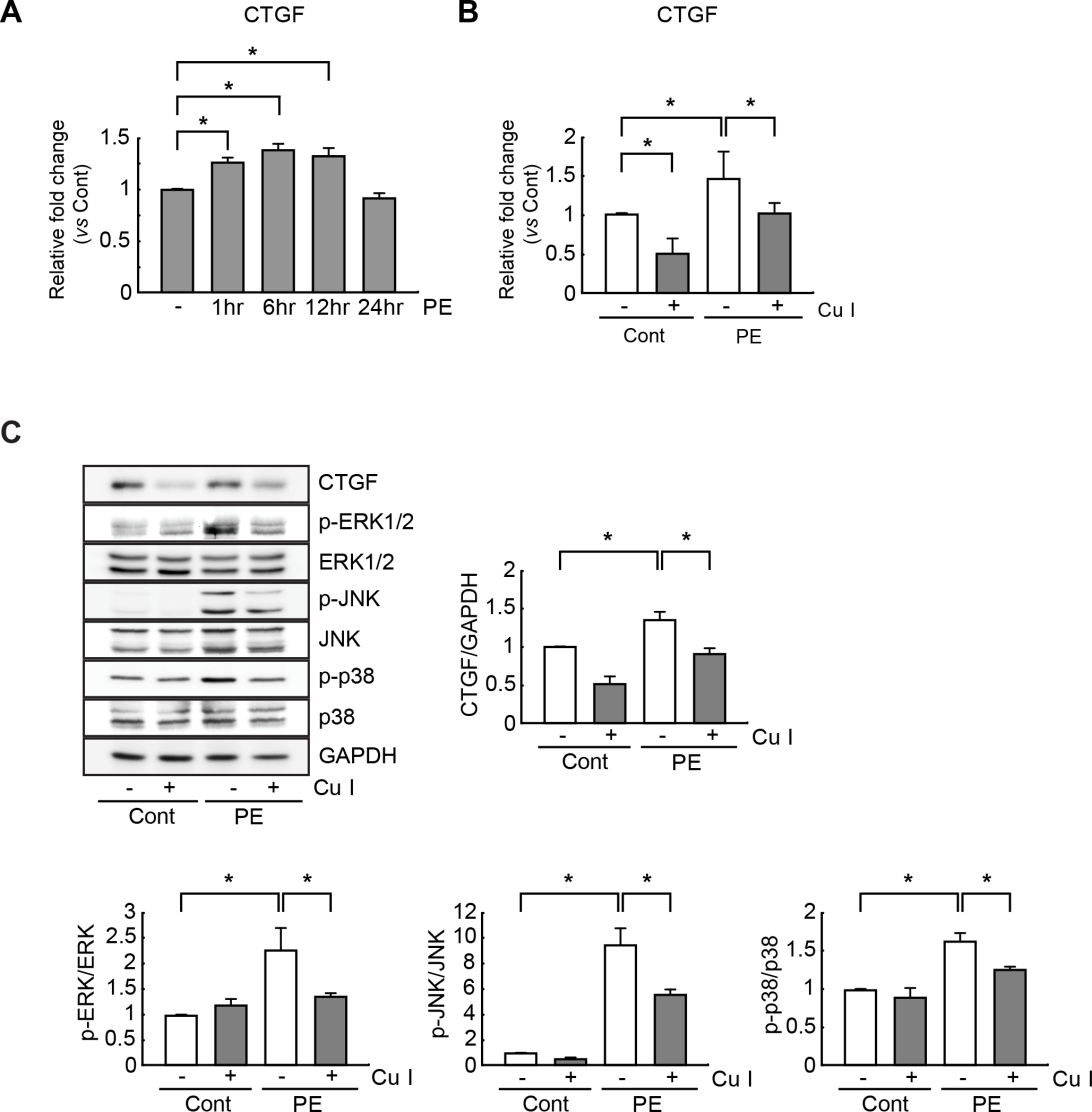
Cucurbitacin I inhibits CTGF expression and MAPK signaling in hypertrophic cardiomyocytes. (A) CTGF mRNA expression levels were measured by quantitative RT-PCR in hypertrophic cardiomyocytes stimulated with PE (100 μM) for 1, 6, 12, and 24 h. (B) Cardiomyocytes were pretreated with cucurbitacin I (1 μM), and then stimulated with PE (100 μM) for 6 h. Next, CTGF mRNA expression levels were measured by quantitative RT-PCR. (C) Cardiomyocyte extracts (50 μg) were subjected to Western blot analysis of CTGF and MAPK, (ERK1/2, JNK, and p38) protein expression levels. The expression levels of CTGF, the MAPKs, and the phosphorylated forms of the MAPKs (p-ERK1/2, p-JNK, and p-p38 kinase) were estimated by measuring band densities with NIH Image J software. GAPDH was used as a loading control, and Western blot analysis was performed in triplicate with three independent samples. Data are expressed as fold changes ± S.D. vs. control group. Significance was measured via a two-way ANOVA. * *P* < 0.05. Cont, control; Cu I, Cucurbitacin I.

Because CTGF is critically involved in the progression of cardiac hypertrophy, we reasoned that cucurbitacin I might exercise its protective actions in cardiomyocytes through modulation of CTGF expression/signaling. To examine the impact of cucurbitacin I on the CTGF expression profile during the hypertrophic process, neonatal rat cardiomyocytes were cultured for 24 h with or without cucurbitacin I (1 μM), incubated with PE (100 μM) for another 6 h, and subjected to quantitative RT-PCR. As a result, CTGF mRNA expression levels were gradually increased in PE-stimulated cardiomyocytes, with the highest level observed at 6 h after the addition of PE without cucurbitacin I (1.5-fold increase vs. vehicle-treated control cells; Fig 3A). This increase was completely blocked by cucurbitacin I pretreatment; moreover, the compound also reduced CTGF mRNA levels in control cells (Fig 3B). Similarly, immunoblot analysis demonstrated that cucurbitacin I downregulated CTGF protein levels in both control and PE-treated cells (Fig 3C).

A previous study indicated that the onset of cardiac hypertrophy critically depends upon activation of MAPKs [26], and recent work demonstrated that CTGF activate numerous MAPKs, including ERK1/2, JNK, and p38 kinases [5]. Therefore, cucurbitacin I might plausibly suppress the activity of these MAPKs in hypertrophic cardiomyocytes. To evaluate this hypothesis, western blot analysis was performed with phospho-specific antibodies against ERK1/2, JNK, and p38 kinase. Phosphorylation of all three MAPKs was significantly augmented in PE-stimulated cardiomyocytes, with 2.2-fold, 9.5-fold, and 1.6-fold increases observed for p-ERK1/2, p-JNK, and p-p38 kinase expression levels, respectively, in-PE-treated vs. vehicle-treated control cells. In line with the observed effects on CTGF content, PE-induced MAPK phosphorylation was dramatically decreased by cucurbitacin I pretreatment (Fig 3C). Hence, cucurbitacin I effectively inhibits the hypertrophy-triggered increase in CTGF content and the ensuing phosphorylation-induced activation of MAPKs in PE-treated cardiomyocytes.

### Cucurbitacin I blocks the TGF- β/Smad fibrotic signaling pathway in hypertrophic cardiomyocytes

**Fig 4:**
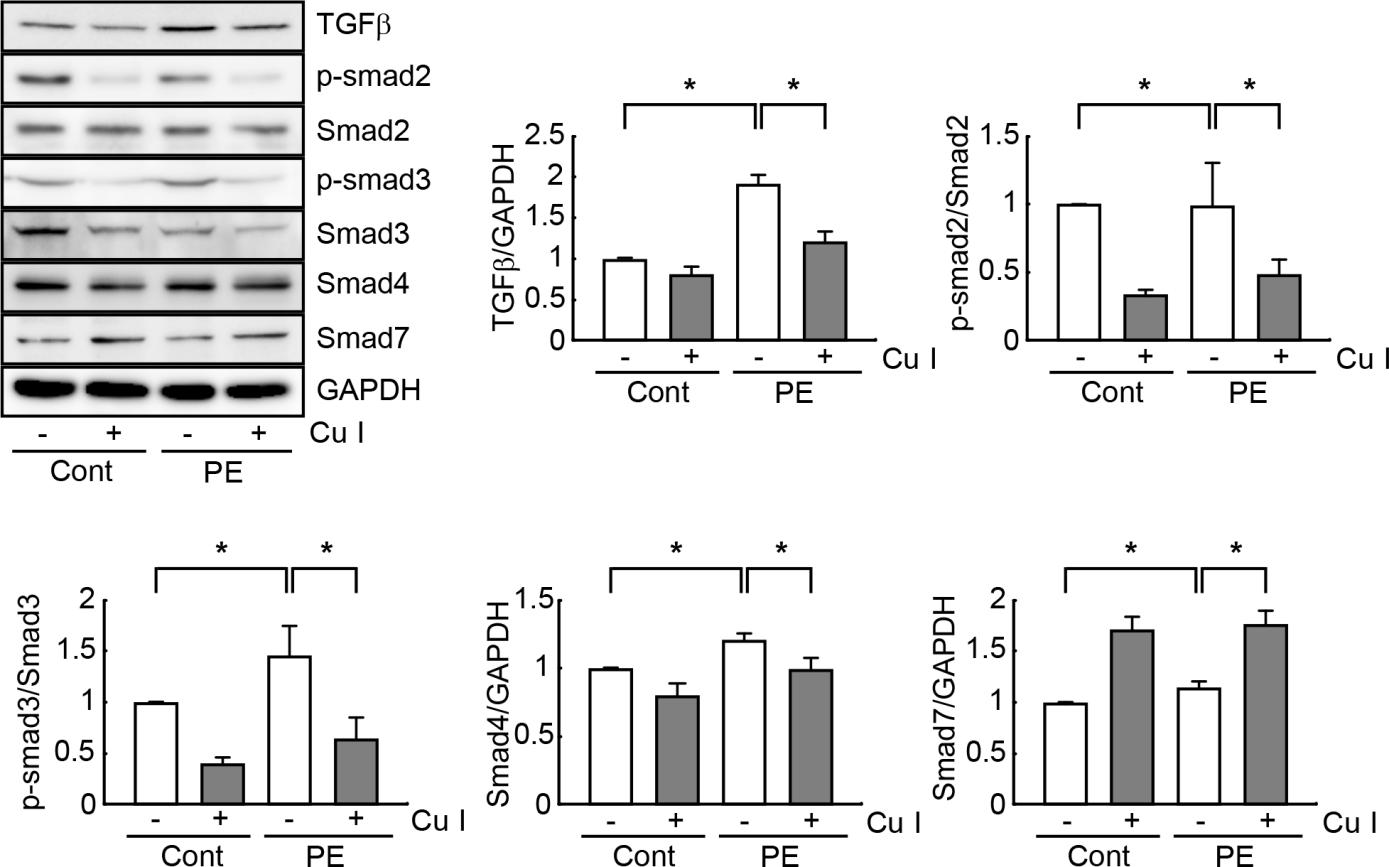
Cucurbitacin I blocks the TGF-β/Smad signaling pathway in hypertrophic cardiomyocytes. Cell extracts (50 μg) were used for Western blot analysis of TGF-β and Smad (Smad2, 3, and 7) protein expression levels. The expression levels of TGF-β, Smad2, 3, and 7, and the phosphorylated forms of Smad2 and 3 (p-Smad2 and p-Smad3) were estimated by measuring band densities with NIH Image J software. GAPDH was used as a loading control. And Western blot analysis was performed in triplicate with three independent samples. Data are expressed as fold changes ± S.D. vs. control group. Significance was measured via a two-way ANOVA. * *P* < 0.05. Cont, control; Cu I, Cucurbitacin I.

TGF-β/Smad signaling pathway reportedly participates in the progression of cardiac hypertrophy and cardiac fibrosis [27]. We therefore investigated the supposition that cucurbitacin I can negatively regulate TGF-β/Smad signaling in PE-treated cardiomyocytes. Western blot analysis revealed that TGF-β expression was 2-fold higher in PE-treated cardiomyocytes relative to control cardiomyocytes. However, the PE-induced increase in TGF-β levels was inhibited by pretreatment with cucurbitacin I (Fig 4). In the same manner, phosphorylation of Smad2 and 3 was significantly upregulated in PE-treated cardiomyocytes, but this induction was prevented by cucurbitacin I pretreatment (Fig 4). By contrast, the expression level of Smad7, as a negative regulator of TGF-β/Smad signaling, was significantly increased in cucurbitacin I-pretreated cardiomyocytes with or without PE treatment, whereas, Smad7 levels remained unchanged in PE-treated cells not receiving cucurbitacin I (Fig 4). These data signify that cucurbitacin I inhibits TGF-β/Smad signaling pathway in hypertrophic cardiomyocytes.

### Anti-hypertrophic actions of cucurbitacin I are blunted in CTGF-silenced or TGF-β-silenced PE-treated cardiomyocytes

**Fig 5:**
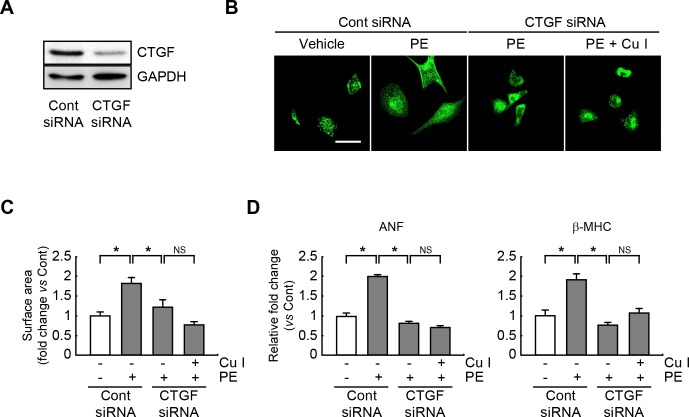
The anti-hypertrophic impact of cucurbitacin I is attenuated in the CTGF-silenced, hypertrophic cardiomyocytes. (A) Expression of CTGF was measured by western blotting in cardiomyocytes transfected with control or CTGF siRNA. (B) Representative photograph of cardiomyocytes after transfection with a scrambled siRNA or CTGF siRNA and pretreatment with cucurbitacin I (1 μM), followed by exposure to PE (100 μM). Sarcomeric organization of the cardiomyocytes was visualized by staining with an anti-α-actinin antibody. (C) Cell surface areas were measured by using NIH Image J software (n = 100 cells). Scale bars, 50 μm. (D) Quantitative RT-PCR analysis of ANF and β-MHC mRNA expression levels in scrambled siRNA- or CTGF siRNA-transfected cardiomyocytes treated with cucurbitacin I and/or PE. All experiments were performed in triplicate with three independent samples. Data are expressed as fold changes ± S.D. vs. control group. Significance was measured via a two-way ANOVA. * *P* < 0.05. scr, scrambled siRNA; si-CTGF, si-RNA against CTGF; Cont, control; Cu I, Cucurbitacin I; NS, not significant.

**Fig 6:**
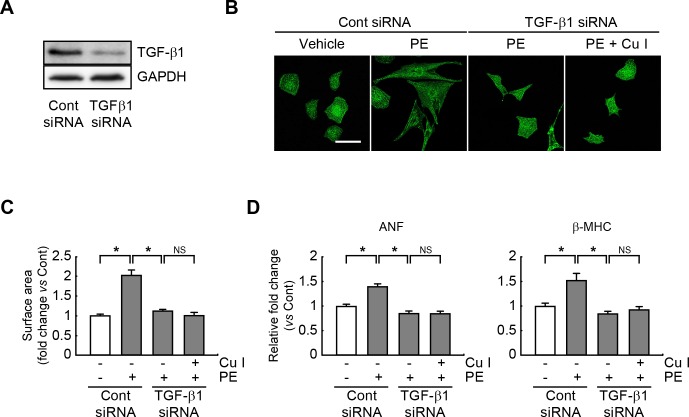
The anti-hypertrophic impact of cucurbitacin I is attenuated in the TGF-β1-silenced, hypertrophic cardiomyocytes. (A) Expression of TGF-β1 was measured by western blotting in cardiomyocytes transfected with control or TGF-β1 siRNA. (B) Representative photograph of cardiomyocytes transfected with a scrambled siRNA or TGF-β1 siRNA, and pretreatment with cucurbitacin I (1 μM), followed by exposure to PE (100 μM). Sarcomeric organization of the cardiomyocytes was visualized by staining with anti-actinin antibody. (C) Cell surface areas were measured by using NIH Image J software (n = 100 cells). Scale bars, 50 μm. (D) Quantitative RT-PCR analysis for ANF and β-MHC expression levels in scrambled siRNA- or TGF-β1 siRNA-transfected cardiomyocytes treated with cucurbitacin I and/or PE. All experiments were performed in triplicate with three independent samples. Data are expressed as fold changes ± S.D. *vs* control. Significance was measured via two-way ANOVA. * *P*<0.05. Cont, control; Cu I, Cucurbitacin I; PE, phenylephrine; NS, not significant.

To further explore whether CTGF signaling is required for the anti-hypertrophic actions of cucurbitacin I, CTGF expression was silenced in neonatal rat cardiomyocytes. Western blot analysis revealed that CTGF expression was decreased when CTGF-siRNA was transfected (Fig 5A). The CTGF-silenced cells were then pretreated with cucurbitacin I and stimulated with PE. CTGF expression was abrogated by transfection of siRNA against CTGF. Consistently, the hypertrophic response (as determined by enlarged cell size and elevated expression of ANF and β-MHC) was significantly diminished in CTGF siRNA-transfected, PE-treated cardiomyocytes compared with scrambled siRNA-transfected, PE-treated cells (Fig 5B, 5C and 5D). Moreover, cucurbitacin I was no longer effective as an anti-hypertrophic agent in CTGF siRNA-transfected, PE-treated cells and failed to reduce cell size (Fig 5B and 5C) or ANF and β-MHC content (Fig 5D). In similar work, the TGF-β-silenced cells were also pretreated with cucurbitacin I and followed by PE stimulation. As a result, the hypertrophic response was significantly inhibited in TGF-β1 siRNA- transfected, PE-treated cells (Fig 6). The anti-hypertrophic actions of cucurbitacin I was also impaired in TGF-β siRNA-transfected, PE-treated cells (Fig 6). These finding suggested that CTGF and TGF-β expressions/signalings, at least in part, mediates the anti-hypertrophic properties of cucurbitacin I in cultured cardiomyocytes.

## Discussion

Cardiac hypertrophy is a major risk factor for cardiovascular morbidity and mortality [28, 29]. Although the disorder is initially considered as compensatory for mechanical loading, prolonged cardiac hypertrophy leads to systolic and diastolic dysfunction and ultimately the development of heart failure [2]. Therefore, intensive efforts have been made to identify negative regulators of cardiac hypertrophy and to illuminate their underlying anti-hypertrophic mechanisms. Indeed, many negative regulators with beneficial actions against cardiac hypertrophy have also been identified over the past decade [30].

Cucurbitacins are natural compounds known for their potent pharmaceutical activities. There are 12 main categories to group cucurbitacins and their derivatives according to their side-chain variations, and they have diverse pharmacological and biological activities according to their different structures. Among cucurbitacin isoforms, cucurbitacin I and B have been intensively investigated for their anti-oxidant, anti-proliferative, and anti-inflammatory effects [31]. Cucurbitacin I is a triterpenoid member of the cucurbitacin family that shows cytotoxic and anti-proliferative activities in several types of cancer cells [32–34]. Nevertheless, its ability to alleviate cardiac disease is unclear. The present study sought to evaluate the pharmacological actions of cucurbitacin I against cardiac hypertrophy in PE-stimulated, cultured rat neonatal cardiomyocytes. At low concentration (1 μM), cucurbitacin I inhibited the hypertrophic response *in vitro*. To the best our knowledge, this is the first indication that cucurbitacin I might exhibit actions against cardiac hypertrophy. As such, it will be interesting to investigate the actions of the other cucurbitacins against in cardiac hypertrophy and fibrosis.

In the heart, CTGF participates in a variety of pathological processes, ranging from the development of cardiac hypertrophy to cardiac fibrosis, and remodeling. The expression of CTGF is elevated in the hypertrophied and failing hearts [35], and exposure of neonatal cardiomyocytes to CTGF can induce cardiac hypertrophy de novo [5]. A recent study showed that the pro-hypertrophic effects of CTGF are associated with the activation of MAPK pathways [5]. In turn, activated MAPKs, including ERK1/2, JNK, and p38 kinase, contribute to the induction of cardiac hypertrophy. Our results demonstrate that cucurbitacin I attenuated CTGF induction and its MAPK signaling in the hypertrophied cardiomyocytes

Cardiac fibrosis is a pathological feature of cardiac hypertrophy and heart failure, and is characterized by interstitial fibroblast proliferation and the deposition of excessive amounts of collagen and other extracellular matrix components [36]. TGF-β and CTGF are key promoters of fibrosis during the development of cardiac hypertrophy [37]. Overexpression of TGF-β in transgenic mice leads to cardiac hypertrophy and fibrosis, whereas blockade of TGF-β with neutralizing antibodies inhibits hypertrophic and fibrotic responses [38, 39]. Indeed, CTGF and TGF-β contribute to the induction of many heart diseases, such as cardiac hypertrophy, myocardial infarction, and cardiac fibrotic diseases in a cooperative manner [6]. For these reasons, the present study focused on CTGF-mediated MAPK and TGF-β/SMAD signaling to elucidate the possible mechanisms of cucurbitacin I-mediated inhibition of cardiac hypertrophy. Our study demonstrates that cucurbitain I could ameliorate cardiac hypertrophy by targeting CTGF/TGF-β and their signaling. Future studies should examine the hypothesis that cucurbitacin I may be effective against other cardiac diseases, such as heart failure, myocardial infarction, and so on.

In conclusion, the current investigation provides new evidence regarding the inhibitory role of cucurbitacin I against myocardial hypertrophy. Furthermore, we demonstrated that these beneficial properties are most likely mediated by suppression of CTGF and its downstream signaling pathways, MAPK and TGF-β/Smad signaling, which are attractive targets for the prevention and/or treatment of cardiac diseases. We further propose that cucurbitacin I might be useful for the treatment and prevention of cardiac hypertrophy and heart failure.
